# Unfolded Coprime Linear Array with Three Subarrays for Non-Gaussian Signals: Configuration Design and DOA Estimation

**DOI:** 10.3390/s22041339

**Published:** 2022-02-10

**Authors:** Meng Yang, Jingming Li, Changbo Ye, Jianfeng Li

**Affiliations:** 1 College of Electronic Information Engineering, Nanjing University of Aeronautics and Astronautics, Nanjing 211106, China; yangmeng19861213@126.com (M.Y.); lijianfeng@nuaa.edu.cn (J.L.); 2 Jiangsu Automation Research Institute, Lianyungang 222000, China; 3 AVIC Shenyang Aircraft Design & Research Institute, Shenyang 110034, China; lijingmingjlu@163.com

**Keywords:** DOA estimation, non-Gaussian signals, coprime linear array, consecutive degree of freedom, discrete Fourier transform

## Abstract

In this paper, we investigate the problem of sparse array design for the direction of the arrival (DOA) of non-Gaussian signals and exploit the unfolded coprime linear array with three subarrays (UCLATS) to obtain physical sensors location. With the motivation from the large consecutive degree of freedom (DOF), we optimize the process of obtaining physical sensors location from two steps. Specifically, the first is to model the process of obtaining the longest consecutive virtual sum co-array from a given number of physical array elements into a global postage-stamp problem (GPSP), whose solution can be employed to determine the locations of the longest possible consecutive sum co-array (2-SC) and initial physical array. The second step is to multiply the location of the virtual sum co-array by appropriate coprime coefficients to generate UCLATS and then multiply the initial physical array position by the same corresponding coefficients to obtain physical sensors location. Besides, an algorithm is proposed to obtain DOA estimates, which employs the discrete Fourier transform (DFT) method and partial spectrum searching multiple signal classification (PSS-MUSIC) algorithm to obtain initial estimates and fine estimates, respectively, termed as the DFT-MUSIC method. Compared with the traditional total spectrum searching MUSIC (TSS-MUSIC) algorithm, the DFT-MUSIC method performs the same asymptotical performance of DOA estimation with less than 10% complex multiplication times, which can be verified by numerical simulations under the same condition.

## 1. Introduction

Array signal processing (ASP) exploits the sensor array to receive spatial signals in order to obtain discrete observation data. Compared with a single directional sensor, the sensor array has many advantages such as stronger spatial gain, more flexible beam control and higher spatial angle resolution; thus, it has been widely used in beamforming, direction of arrival (DOA) estimation, source separation and microphone arrays for acoustic imaging over the decades [1,2,3,4,5]. Especially, DOA estimation plays an important role in array signal processing, which is widely applied in wireless communication system, radar system and navigation [1,6]. Conventional DOA estimation methods mainly focus on uniform linear array (ULA) [7,8] or uniform planar array (UPA) for their simple and symmetric structure, whose inter-element spacing is required to be no longer than half wavelength to avoid angle ambiguity, which results in limited DOA estimation accuracy.

To further improve the performance of DOA estimation, various sparse geometries are proposed to achieve an extended array aperture, enhanced degree of freedom (DOF) and reduced mutual coupling, whose typical representatives include coprime array (CA) [9,10,11,12], nested array (NA) [11], and minimum redundancy array (MRA) [13]. These sparse arrays are designed based on the SOC under the assumption that the sources are Gaussian, whose virtual elements can be obtained from the sum co-array (2-SC) or difference co-array (2-DC) of physical sensors. Nevertheless, the 2-DC of CA generates many holes that decrease consecutive DOF significantly; the dense part of NA results in serious mutual coupling. To tackle these problems, scholars propose NA-based and CA-based arrays, such as augmented coprime array (ACA) [14], augmented nested array (ANA) [15], unfolded coprime linear array (UCLA) [16,17], coprime array with displaced subarrays (CADiS) [18], generalized nested array (GNA) [19], and so on.

Note that all sparse array geometries mentioned above are designed for Gaussian sources, while most signals are non-Gaussian in engineering applications; hence, it is necessary to design a sparse structure for non-Gaussian sources, which employs fourth-order cumulant (FOC) methods to obtain DOA estimates. FOC methods are not sensitive to noise, which can be completely suppressed whether the noise is white or colored [20,21], such as MUSIC-LIKE [22,23] and virtual-ESPRIT [24,25] algorithm. The virtual co-arrays employed by vectorized FOC methods can be obtained from fourth-order difference co-array (FODC) or difference co-array of sum co-array (2-DCSC) operation of physical sensors. Reference [26] proposed the concept of multiple-level NA, and fourth-level NA (FL-NA) is introduced in detail, which generated array geometry with improved consecutive DOF (cDOF) performance from 2q-th order cumulant perspective. Based on FL-NA, reference [27] proposed Enhanced Fourth-Level Nested Array (E-FL-NA) with larger cDOF than FL-NA under the same conditions, but it still suffers from serious mutual coupling because of the inter-element spacing of the first subarray. In addition, reference [28] proposed sparse array with FODC enhancement based on CPA (SAFE-CPA), which constructed a physical sensors structure by adding another subarray. Expanding and Shift scheme NA-NA (EAS-NA-NA) and EAS-NA-CPA scheme were designed in [29], which increased the consecutive lags greatly because of multiple subarrays. These sparse arrays are designed based on the multiple subarrays constructed as CA or NA, which optimize array configuration locally rather than as a whole; besides, all the subarrays configured as CA or NA compulsorily further limits the performance of cDOF.

In this paper, a sparse array design method based on the global postage-stamp problem (GPSP) [30,31,32] and unfolded coprime linear array with three subarrays (UCLATS) is proposed for non-Gaussian signals, which is motivated by a large number of cDOF and further elaborated by a given sparse array geometry design example. The process of optimizing physical sensors location can be divided into two steps; specifically, the first step is to model the problem of obtaining the longest possible consecutive virtual 2-SC from a given number of physical array elements into a GPSP and then utilize the solution of the GPSP to obtain the position of the virtual 2-SC and the position of the initial physical array. The second step is to multiply the positions of virtual 2-SC by appropriate coefficients to generate UCLATS. Consequently, the position of physical sensors can be obtained by multiplying the corresponding coefficient to the initial physical array. An example is given to illustrate the process of sparse array design. Besides, an algorithm is proposed, where discrete Fourier transform (DFT) [33] algorithm is utilized to obtain initial DOA estimates, and partial spectrum searching MUSIC (PSS-MUSIC) algorithm is employed to obtain accuracy DOA estimates, termed as DFT-MUSIC. The proposed method achieves better DOA estimation performance than DFT method—the same asymptotical performance of DOA estimation as TSS-MUSIC algorithm with less than 10% complex multiplication times.

Specifically, the contributions can be summarized as follows:

1. We design the structure of UCLATS, whose cDOF of 2-DC is also provided. The problem of sparse array design with non-Gaussian signals is investigated from GPSP perspective and the structure of UCLATS.
2. We divide the process of obtaining the location of physical sensors into two steps, which optimizes the array location step by step, resulting in lower design difficulty.
3. We devise DFT-MUSIC algorithm for a better balance between computational complexity and DOA estimation performance, which can be utilized for the non-Gaussian signals with the proposed array geometry.

In Section 2, we introduce 2-DC, 2-SC, 2-DCSC, FODC and the properties of UCLATS. Section 3 describes the GPSP and the procedure of proposed sparse array geometry design principle. Section 4 presents the DFT-MUSIC method. Section 5 shows the performance analysis. Section 6 elaborates the simulation results, and Section 7 draws the conclusions.

Notations: Matrices and vectors are denoted by bold lowercase and uppercase characters, respectively. ⊗ and ⊙ represent the Kronecker product and Khatri-Rao product, respectively. ${\left(\cdot \right)}^{T}$, ${\left(\cdot \right)}^{H}$ and ${\left(\cdot \right)}^{*}$ stand for the transpose, conjugate transpose and complex conjugation operation, respectively. vec(⋅) means the vectorization operation of a matrix. Cum(⋅) represents the cumulant operator and round(⋅) is the rounding operation.

## 2. Preliminaries

In this part, 2-DC, 2-SC, 2-DCSC, FODC, the properties of UCLATS and the received signal model are introduced.

### 2.1. 2-DC, 2-SC, 2-DCSC, FODC

**Definition** **1.** *The 2-DC location*D*can be defined as* [11]

(1) $$\begin{array}{ll} D & =D^{+}\cup D^{-}=D_{self}\cup D_{cross}\\ & =\{d_{i}-d_{j},d_{i},d_{j}\in S\} \end{array}$$

*where* $D^{+}$ *and* $D^{-}$ *represent the positive and negative elements of* D *, respectively.* $D_{self}$ *and* $D_{cross}$ *are self-difference co-array set and cross-difference co-array set, respectively.* S *denotes the physical sensors location set.*

**Definition** **2.** *The 2-SC location*$S_{s}$*can be defined as* [34]

(2) $$\begin{array}{ll} S_{s} & =S_{self}\cup S_{cross}\\ & =\{d_{i}+d_{j},d_{i},d_{j}\in S\} \end{array}$$

*where* $S_{self}$ *and* $S_{cross}$ *denote self-sum co-array set and cross-sum co-array set, respectively.*

**Definition** **3.** *The 2-DCSC location*$D_{dcsc}$*can be defined as* [34]

(3) $$\begin{array}{l} D_{dcsc}=\{d_{i}-d_{j},d_{i},d_{j}\in D\}\\ =\{(d_{i1}+d_{j2})-(d_{j1}+d_{i2}),d_{i1},d_{i2},d_{j1},d_{j2}\in S\} \end{array}$$

*where* S *and* D *represent the physical sensors and 2-DC location set, respectively.*

**Definition** **4.** *The FODC location*$D_{4dc}$*can be defined as* [26,28]

(4) $$\begin{array}{l} D_{FODC}=\{d_{i}-d_{j},d_{i},d_{j}\in D\}\\ =\{(d_{i1}-d_{i2})-(d_{j1}-d_{j2}),d_{i1},d_{i2},d_{j1},d_{j2}\in S\}\\ =\{(d_{i1}+d_{j2})-(d_{j1}+d_{i2}),d_{i1},d_{i2},d_{j1},d_{j2}\in S\} \end{array}$$

*where* S *and* D *represent the physical sensors and 2-DC location set, respectively. Therefore, it can be concluded that the 2-DCSC and FODC of physical arrays can be transformed to each other from (3) and (4).*

### 2.2. The Properties of UCLATS

**Figure 1:**
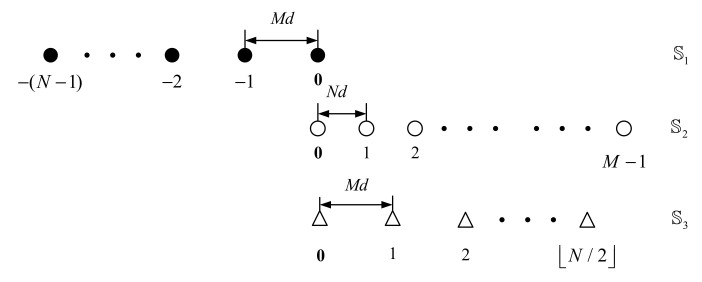
Unfolded coprime linear array with three subarrays (UCLATS).

The configuration of UCLATS has been shown in Figure 1, which contains three sparse uniform subarrays overlapped at the origin. The position of UCLATS can be given by

(5) $$\left\{\begin{array}{l} S_{1}=\langle -(N-1),0\rangle Md\\ S_{2}=\langle 0,M-1\rangle Nd\\ S_{3}=\langle 0,\left\lfloor N/2\right\rfloor \rangle Md \end{array}\right.$$

(6) $$S_{UCLATS}=S_{1}\cup S_{2}\cup S_{3}$$

where M and N are coprime integers, which represent the physical sensors of subarray 1 $S_{1}$ and subarray 2 $S_{2}$, respectively. d=λ/2 and λ denote wavelength. In addition, the total physical sensors number of UCLATS is $T=M+N+\left\lfloor N/2\right\rfloor -1$.

**Lemma** **1.** *The following properties hold for UCLATS:*

*(a)*  *The DOF of UCLATS has been presented in* Table 1 *with different combinations of M and N.*
*(b)*  $D_{UCLATS}$*contains the consecutive lags in the range of*[−(MN+N−1)d,(MN+N−1)d]*with inter-element spacing*d*, where the positive consecutive lags of cross-difference co-array*$D_{cUCLATS}$*are distributed in range*[0,(MN−1)d]*and*[(MN+1)d,(MN+N−1)d].

**Proof.**  See Appendix A. □

### 2.3. Received Signal Model

Assume that there are K uncorrelated far-field narrowband non-Gaussian signals impinging on a linear array with DOAs $(\theta_{k},k=1,2,\ldots ,K),$ where $\theta_{k}$ denotes the *k*-th source. The location set of arbitrary linear array with L sensors is $S=\{d_{1},d_{2},\cdots ,d_{L}\}$, the received signal x(t) can be modeled as [19]

(7) x(t)=As(t)+n(t)

where $A(\theta )=[a(\theta_{1}),a(\theta_{2}),\cdots ,a(\theta_{K})]\in {\mathbb{C}}^{L\times K}$ denotes the steering matrix, and $a(\theta_{k})={\left[e^{-j2\pi d_{1}\sin(\theta_{k})/\lambda },e^{-j2\pi d_{2}\sin(\theta_{k})/\lambda },\cdots ,e^{-j2\pi d_{L}\sin(\theta_{k})/\lambda }\right]}^{T}\in {\mathbb{C}}^{L\times 1}$ is the steering vector. $s(t)={\left[s_{1}(t),s_{2}(t),\cdots ,s_{K}(t)\right]}^{T}\in {\mathbb{C}}^{K\times 1},1\leq t\leq J$ is the non-Gaussian signals matrix, where J is the number of snapshots, $n(t)\in {\mathbb{C}}^{L\times 1}$ is the additive Gaussian noise vector.

The FOC matrix of x(t) can be represented by [24,25]

(8) $$\begin{array}{cc} C_{4,x} & =\sum_{k=1}^{K}c_{4,s_{k}}[a(\theta_{k})⊗a^{*}(\theta_{k})]{\left[a(\theta_{k})⊗a^{*}(\theta_{k})\right]}^{H}\\ & =\sum_{k=1}^{K}c_{4,s_{k}}a_{4,x}(\theta_{k})a_{4,x}^{H}(\theta_{k}) \end{array}$$

where $c_{4,s_{k}}=Cum(s_{k}(t),s_{k}(t),s_{k}^{*}(t),s_{k}^{*}(t))$ is the FOC of $s_{k}(t)$, and

(9) $$a_{4,x}(\theta_{k})=a(\theta_{k})⊗a^{*}(\theta_{k}),1\leq k\leq K$$

whose elements are with the specific form $e^{-j\pi (d_{i}-d_{j})\sin\theta_{k}},1\leq i,j\leq L$, which can be constructed by $a(\theta_{k})$.

To obtain the vectorized signal model, we reconstruct $C_{4,x}$ as [34]

(10) $$z=vec(C_{4,x})=A_{vec}(\theta )p$$

where

(11) $$\begin{array}{cc} A_{vec}(\theta ) & =[a_{4,x}^{*}(\theta_{1})⊗a_{4,x}(\theta_{1}),a_{4,x}^{*}(\theta_{2})⊗a_{4,x}(\theta_{2}),\cdots ,a_{4,x}^{*}(\theta_{K})⊗a_{4,x}(\theta_{K})]\\ & =[a_{vec}(\theta_{1}),a_{vec}(\theta_{2}),\cdots ,a_{vec}(\theta_{K})] \end{array}$$

whose elements are constructed by $a_{4,x}(\theta_{k})$, and $p={\left[c_{4,s_{1}},c_{4,s_{2}},\cdots ,c_{4,s_{K}}\right]}^{T}$. 

## 3. Sparse Array Design Principle

Motivated from the advantages of larger cDOF generated by the 2-DCSC of physical array, according to Equation (4) and [30], the process of obtaining 2-DCSC of physical sensors can be optimized step by step. Specifically, the first is to model the process of obtaining the consecutive longest 2-SC from given number as a GPSP, whose solution can be utilized to determine the locations of initial physical arrays and virtual 2-SC. The second is to multiply virtual 2-SC by appropriate coprime coefficients to form UCLATS, consequently, the physical array location can be obtained by multiplying the corresponding coprime coefficients to initial physical arrays location.

### 3.1. Global Postage-Stamp Problem (GPSP)

According to [31,32], we describe the GPSP as: for given positive integers h and k, a set with k non-negative integers can be given by

(12) $$S_{k}=\{0=a_{1}<\cdots <a_{k}\}$$

**Remark** **1.** *The elements in*$S_{k}$*should be summed h times to achieve the consecutive integers range with*$0,1,2,\cdots ,n_{h}(S_{k})$.

**Remark** **2.**
$n_{h}(S_{k})$ *needs to be as large as possible.*

The solutions to the GPSP have been given in [30,31,32]; we also present the results shown in Table 2.

### 3.2. Sparse Array Design Principle Based on UCLATS

Suppose that the physical sensors are composed of three sparse linear subarrays with *M*, *N* and $\left\lfloor N/2\right\rfloor +1(N<M)$ elements, respectively. According to Section 3.1, the sets $S_{N}$, $S_{M}$ and $S_{\left\lfloor N/2\right\rfloor }$ can be obtained from the solution to GPSP. For $S_{N}$, if all elements are negative, the consecutive integers set that can be obtained from *N* sensors are

(13) $$\left\{-n_{2}(S_{N}),\cdots ,-2,-1,0\right\}$$

where $S_{N}$ represents a set with *N* integers, $n_{2}(S_{N})$ is the largest possible integer mentioned in (12), which can be obtained by Table 2. For $S_{M}$ and $S_{\left\lfloor N/2\right\rfloor }$, if all elements are positive, the consecutive integers set which can be obtained from *M* and $\left\lfloor N/2\right\rfloor +1$ sensors are

(14) $$\begin{array}{l} \left\{0,1,2,\cdots ,n_{2}(S_{M})\right\}\\ \left\{0,1,2,\cdots ,n_{2}(S_{\left\lfloor N/2\right\rfloor })\right\} \end{array}$$

The 2-SC of $S_{N}$, $S_{M}$ and $S_{\left\lfloor N/2\right\rfloor }$ can be associated with three co-subarrays of UCLATS if we multiply Equations (13) and (14) by appropriate coprime coefficients. The co-subarray 1 of UCLATS associated with (13) can be denoted as

(15) $$S_{sN}=\{-n_{2}(S_{N})d_{1},\cdots ,-2d_{1},-d_{1},0\}$$

where $d_{1}=(n_{2}(S_{M})+1)d$ represents the element spacing of co-subarray 1 and co-subarray 3, d=λ/2 and λ is wavelength. The co-subarray 2 and co-subarray 3 location sets associated with (14) can be expressed as

(16) $$\begin{array}{l} S_{sM}=\{0,d_{2},2d_{2},\cdots ,n_{2}(S_{M})d_{2}\}\\ S_{s\left\lfloor N/2\right\rfloor }=\{0,d_{1},2d_{1},\cdots ,n_{2}(S_{\left\lfloor N/2\right\rfloor })d_{1}\} \end{array}$$

where $d_{2}=(n_{2}(S_{N})+1)d$ represents the element spacing of co-subarray 2. Besides, $n_{2}(S_{M})+1$ and $n_{2}(S_{N})+1$ are coprime integers.

The co-array $S_{sc}=S_{sM}\cup S_{sN}\cup S_{s\left\lfloor N/2\right\rfloor }$ can be considered as UCLATS. Considering that the 2-SC of $S_{N}$, $S_{M}$ and $S_{\left\lfloor N/2\right\rfloor }$ have been multiplied by $d_{1}$ or $d_{2}$, then the location of physical sensors should also be adjusted by multiplication operations. We give the location set of physical sensors as follows,

(17) $$S=S_{N}d_{1}\cup S_{M}d_{2}\cup S_{\left\lfloor N/2\right\rfloor }d_{1}$$

**Figure 2:**
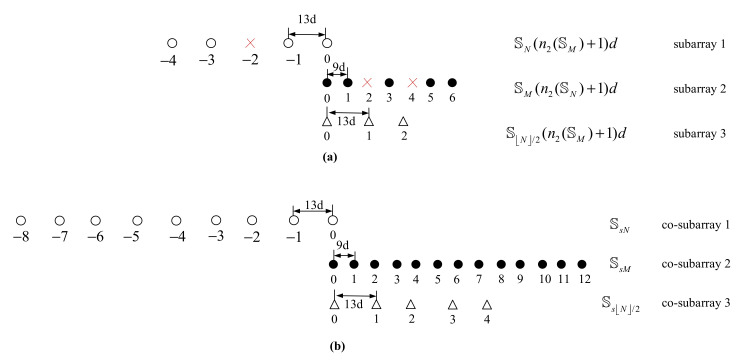
(**a**) Physical sensors location S; (**b**) co-array $S_{sc}=S_{sM}\cup S_{sN}\cup S_{s\left\lfloor N/2\right\rfloor }$.

Figure 2 shows the example of the process mentioned above, where M=5,N=4 $S_{N}=\{-4,-3,-1,0\}$, $S_{M}=\{0,1,3,5,6\}$, $S_{\left\lfloor N/2\right\rfloor }=\{0,1,2\}$. $n_{2}(S_{M})=12,n_{2}(S_{N})=8,n_{2}(S_{\left\lfloor N/2\right\rfloor })=4$, $S_{sM}=9d\times \{-12,\cdots ,-1,0\}$, $S_{sN}=13d\times \{0,1,\cdots ,8\}$, $S_{s\left\lfloor N/2\right\rfloor }=13d\times \{0,1,2,3,4\}$.

## 4. DOA Estimation Method

Conventional MUSIC methods cost expensive computational complexity due to the TSS process. In this section, we propose a DFT-MUSIC method for the proposed array geometry. Specifically, the DFT [33] method for the consecutive 2-DCSC part of the proposed array geometry is utilized to obtain the initial DOA estimates. Subsequently, the PSS-MUSIC algorithm is exploited to obtain the fine DOA estimates.

### 4.1. Initial Estimation by DFT Algorithm

According to [33], the DFT is a non-parametric spectrum analysis method whose DOA estimation performance depends on the number of consecutive virtual elements. Based on Section 2.2, we have illustrated that the 2-DC of UCLATS contains consecutive co-arrays; besides, the relationship between the 2-DCSC of S and the 2-DC of $S_{sc}$ has been also provided. Thus, the consecutive virtual elements part of proposed array geometry with range [−Wd,Wd] can be employed by DFT method to obtain initial estimation, whose total number is T=2W+1, the value of W has been given in (28).

Define the steering vector $a_{c}(\theta_{k})\in {\mathbb{C}}^{T\times 1}$ as

(18) $$a_{c}(\theta_{k})={\left[e^{-j(-W)\pi \sin\theta_{k}},\cdots 1,\cdots ,e^{-jW\pi \sin\theta_{k}}\right]}^{T}$$

Define the normalized DFT matrix $F\in {\mathbb{C}}^{T\times T}$, whose element corresponding to coordinate (p,q) can be expressed as

(19) $${\left[F\right]}_{pq}=\frac{1}{\sqrt{T}}e^{-j\frac{2\pi }{T}pq}$$

Consequently, the new normalized steering vector ${\tilde{a}}_{c}(\theta_{k})$ constructed by F and $a_{c}(\theta_{k})$ can be written as

(20) $${\tilde{a}}_{c}(\theta_{k})=Fa_{c}(\theta_{k})$$

whose q-th element is

(21) $${\left[{\tilde{a}}_{c}(\theta_{k})\right]}_{q}=\frac{1}{\sqrt{T}}\frac{\sin\left[\frac{T}{2}(\frac{2\pi }{T}q-\pi \sin\theta_{k})\right]}{\sin\left[\frac{1}{2}\left(\frac{2\pi }{T}q-\pi \sin\theta_{k}\right)\right]}e^{-j\frac{T-1}{2}\left(\frac{2\pi }{T}q-\pi \sin\theta_{k}\right)}$$

The analysis of the value in Equation (21) has been given in Appendix B, which can be concluded from two aspects. Specifically, when $T\sin\theta_{k}/2=q_{k}$ is an integer, only the value of $q=q_{k}$-th element of ${\tilde{a}}_{c}(\theta_{k})$ is not zero, when $q_{k}$ is not an integer, most elements in (21) are small values close to zero, except the $q=round\left\{q_{k}\right\}$-th element.

Therefore, the initial estimates of $\theta_{k}$ can be obtained by finding the approximate position of the non-zero elements in ${\tilde{a}}_{c}(\theta_{k})$, it can also be used to obtain the number of sources. In applications, the source estimates can be obtained by performing DFT on the vectorized received signal $z_{1}$, which is obtained by sorting and removing the redundancy operation of z. Further, the vector after DFT is $y_{ini}=Fz_{1}$, whose spectrum corresponds to K largest peaks are marked as $q_{k}^{ini}\left(k=1,2,\cdots ,K\right)$; then, the initial DOA estimates can be calculated by

(22) $$\theta_{k}^{ini}=\arcsin(2q_{k}^{ini}/T),\;k=1,2,\cdots ,K$$

### 4.2. Fine Estimation by PSS-MUSIC Method

**Figure 3:**
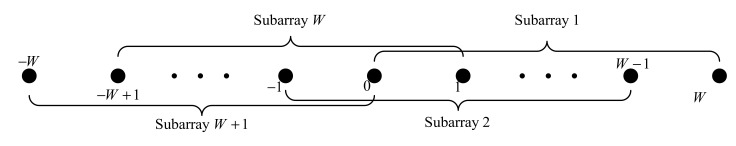
The process of spatial smoothing.

According to Appendix B, when $q_{k}=T\sin\theta_{k}/2$ is not an integer, more accurate DOA estimates cannot be obtained by $q_{k}^{ini}$; thus, PSS-MUSIC is introduced to tackle this problem. Generally, MUSIC methods achieve improved DOA estimation performance, but TSS results in expensive computational complexity. The DFT-MUSIC utilizes PSS under known initial estimates, which reduces complexity effectively. This part first utilizes the DFT method described in Section 4.1 to obtain the initial estimates and then employs the PSS-MUSIC method to obtain fine estimation. The process of spatial smoothing with T consecutive co-arrays is depicted in Figure 3, where T=2W+1, and the value of W is shown in (28).

According to [35], spatial smoothing methods require a full rank FOC matrix. By employing spatial smoothing process, the consecutive co-array steering vector $a_{c}(\theta_{k})$ shown in Equation (18) is divided into W+1 sub-arrays, and each sub-array contains W+1 elements, where the position of *i*-th(1≤i≤W+1) subarray can be denoted by

(23) $$\left\{\left(-i+1+n\right)d,\;n=0,1,\cdots ,W\right\}$$

whose vectorized FOC matrix $z_{1i}$ is constructed within row W+1−i to 2W+1−i of $z_{1}$. The covariance matrix can be constructed by

(24) $$R_{i}=z_{1i}z_{1i}^{H}$$

Sum the covariance matrices of all W+1 subarrays, whose mean value corresponding to full rank spatial smoothing matrix can be calculated by

(25) $$R=\frac{2}{T+1}\sum_{i=1}^{\left(T+1\right)/2}R_{i}$$

Based on the eigenvalue decomposition (EVD), R can be decomposed as

(26) $$R=E_{s}D_{s}E_{s}^{H}+E_{n}D_{n}E_{n}^{H}$$

where $E_{s}$ is formed by the eigenvectors corresponding to the maximum *K* eigenvalues, and $E_{n}$ is composed of the rest eigenvectors. $D_{s}$ and $D_{n}$ are diagonal matrices, the diagonal elements of $D_{s}$ are made up of the largest *K* eigenvalues, and the diagonal elements of $D_{n}$ are composed of other eigenvalues.

Based on the orthogonal relationship between the noise space $E_{n}$ and the steering vector, the spectrum function of the fine estimation in the range $[\theta_{k}^{ini}-\Delta ,\theta_{k}^{ini}+\Delta ],\Delta =1^{\circ }$ can be represented by

(27) $$P_{DFT-MUSIC}(\theta )=\frac{1}{a_{sub}^{H}(\theta )E_{n}E_{n}^{H}a_{sub}(\theta )}$$

where $a_{sub}\left(\theta \right)$ is the steering vector of subarray i(1≤i≤W+1) described in Figure 3, which can be set as $a_{sub}\left(\theta \right)={\left[1,e^{-j2\pi d\sin\theta /\lambda },\cdots ,e^{-j\pi Wd\sin\theta /\lambda }\right]}^{T},\theta \in [\theta_{k}^{ini}-\Delta ,\theta_{k}^{ini}+\Delta ]$. Thus, the fine estimates ${\hat{\theta }}_{k},(k=1,\cdots ,K)$ can be obtained by performing PSS via (27). 

## 5. Performance Analysis

In this section, we compare the performance of the proposed array geometry and DFT-MUSIC method with other arrays and algorithms from the viewpoints of DOF and computational complexity.

### 5.1. Achievable Consecutive DOF

**Figure 4:**
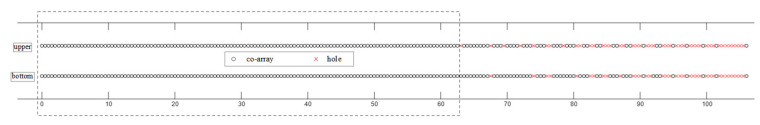
Consecutive DOF comparison.

It is noteworthy to analyze the cDOF of the proposed array. Considering that the $S_{sM}$, $S_{sN}$ and $S_{s\left\lfloor N/2\right\rfloor }$ have been associated with the co-subarrays of UCLATS in Figure 2, according to Lemma 1, the cDOF of the 2-DC of $S_{sc}$ can be obtained by

(28) $$cDOF_{UCLATS}=2W+1$$

which contains consecutive lags in the range of [−Wd,Wd],$W=(n_{2}(S_{M})+2)(n_{2}(S_{N})+1)-1$. Definition 1 points out that the location of 2-DC is symmetric about the origin; thus, we give only the non-negative part of the 2-DC of $S_{sc}$, as depicted in the upper part of Figure 4.

However, for the proposed array, the following relationship is established:

(29) $$cDOF_{proposed}\geq cDOF_{UCLATS}$$

where $cDOF_{proposed}$ denotes the cDOF of proposed array geometry, which can be calculated by the 2-DCSC or FODC of physical sensors. 

As mentioned in Definition 1 and Definition 2, the 2-DC are composed of self-difference co-array and cross-difference co-array, and the 2-SC is composed of self-sum co-array and cross-sum co-array. Considering that the processes of obtaining three subarrays of proposed geometry are separate, the cross-sum co-array of proposed geometry is ignored. Consequently, the 2-SC of $S_{s}$ contains $S_{sc}$ completely. We also give the non-negative part of 2-DCSC of the proposed array geometry, as shown in the bottom part of Figure 4. It is obvious that Equation (29) is established. Besides, the consecutive range of $cDOF_{UCLATS}$ is also contained by $cDOF_{proposed}$. 

**Figure 5:**
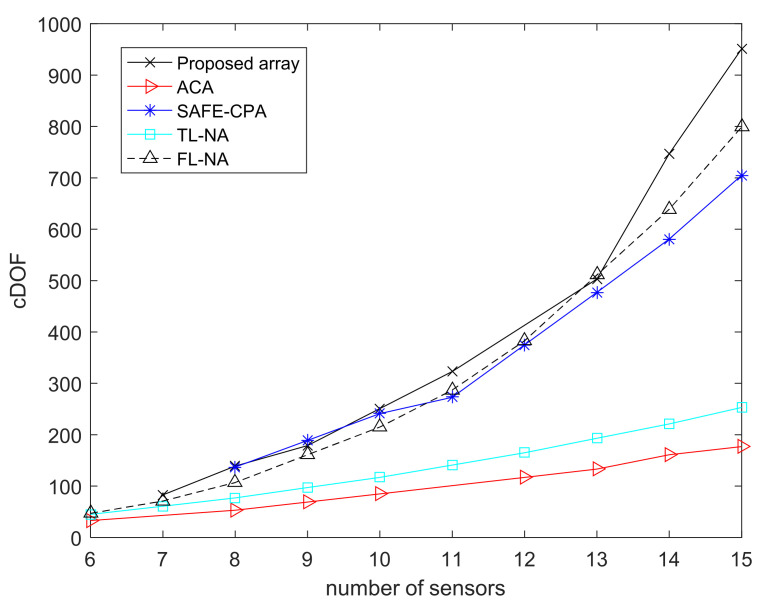
cDOF comparisons of different arrays.

The cDOF comparisons of ACA [14], SAFE-CPA [28], TL-NA [11], FL-NA [26] and proposed array geometry are depicted in Figure 5. Considering that the SOC-based array cannot exploit the properties of 2-DCSC adequately, the proposed array geometry, SAFE-CPA and FL-NA achieve better cDOF performance than TL-NA and ACA. Besides, the proposed array geometry performs better than SAFE-CPA and FL-NA, as the total number of sensors increases. In the case of the same number of sensors, different combinations of subarrays may produce different cDOF; we also present the largest possible combinations of cDOF for each array structure, as shown in Table 3, Table 4 and Table 5.

**Remark** **3.** *The cDOF of the proposed array geometry listed in*Table 4 and Table 5 *is the minimum cDOF, which can be calculated from* $cDOF_{UCLATS}$*, as shown in (28). Considering the existence of the sum co-array between the sub-arrays, the actual cDOF will be a bit larger. e.g., (*T=10*, actual cDOF = 269 > 251).*

### 5.2. Computational Complexity

For non-Gaussian signals, we propose a DFT-MUSIC method for the proposed geometry described in Section 4, which reduces computational complexity of MUSIC algorithm by shrinking the range of spectrum searching. The complexity comparison of TSS-MUSIC algorithm and DFT-MUSIC are listed to verify the efficiency, which can be calculated by the times of complex multiplications. For DFT-MUSIC method, calculating the FOC matrix requires $O\{J{\left(M+N+\left\lfloor N/2\right\rfloor \right)}^{4}\}$, obtaining initial estimates needs $O\{T^{2}\}$, and PSS process costs O{nW(2(W−K)+1)}, where *n* and *T* represent the PSS times and the number of cDOF, respectively. Besides, W=(T+1)/2. For the TSS-MUSIC method, the computational complexity can be calculated by $O\{J{\left(M+N+\left\lfloor N/2\right\rfloor -1\right)}^{4}+W^{3}+n_{1}W(2(W-K)+1)\}$, where $n_{1}$ represents the TSS times.

The subarrays sensors of the proposed array are set to be M=5,N=4, snapshots J=1000, sources number K=2. The step of searching grid is $\Delta =0.01^{\circ }$. Thus, the searching times of TSS and PSS are $n=(2^{\circ }/\Delta )K=400,n=(180^{\circ }/\Delta )=18,000$. According to the discussion mentioned above, the spectrum searching range of DFT-MUSIC and TSS-MUSIC methods are $(\theta_{k}^{ini}-1^{\circ },\theta_{k}^{ini}+1^{\circ }),k=1,\cdots K$ and $(-90^{\circ },90^{\circ }),$ respectively.

The value of the complex multiplications times has been given in Table 6. Because the DFT-MUSIC method performs PSS rather than TSS, it narrows the searching range effectively. Hence, the computational complexity can be reduced effectively, which means that the DFT-MUSIC is computationally efficient.

## 6. Simulations Results

In this section, we exploit 500 Monte Carlo simulations via the DFT-MUSIC algorithm to validate the performance of the proposed array geometry. The root mean square error (RMSE) is given by

(30) $$\mathrm{RMSE}=\frac{1}{K}\sum_{k=1}^{K}\sqrt{\frac{1}{500}\sum_{i=1}^{500}{\left({\hat{\theta }}_{k,i}-\theta_{k}\right)}^{2}}$$

where $\theta_{k}$ represents the elevation of *k*-th target, ${\hat{\theta }}_{k,i}$ is estimates of $\theta_{k}$ in the *i*-th (i=1,…,500) simulation. Besides, the searching interval of DFT-MUSIC algorithm is $\Delta =0.01^{\circ }$.

Suppose that there are *K* uncorrelated far-field non-Gaussian sources with $\theta =[5^{\circ },40^{\circ }]$ incident on different arrays listed in Table 4. The locations of different arrays are shown as follows, TL-NA:{0.5,1,1.5,12,2.5,3,6,9,12,15}, SAFE-CPA:{0,1,1.5,2,3,4.5,17.5,30.5,43.5,56.5}, FL-NA:{0.5,1,1.5,2,4,6,12,18,36,54}, ACA:{0,1.5,2.5,3,4.5,5,6,7.5,10,12.5}, proposed array geometry:{−26,−19.5,−6.5,0,4.5,6.5,13,13.5,22.5,27}.

### 6.1. RMSE Performance Comparison versus Snapshots

**Figure 6:**
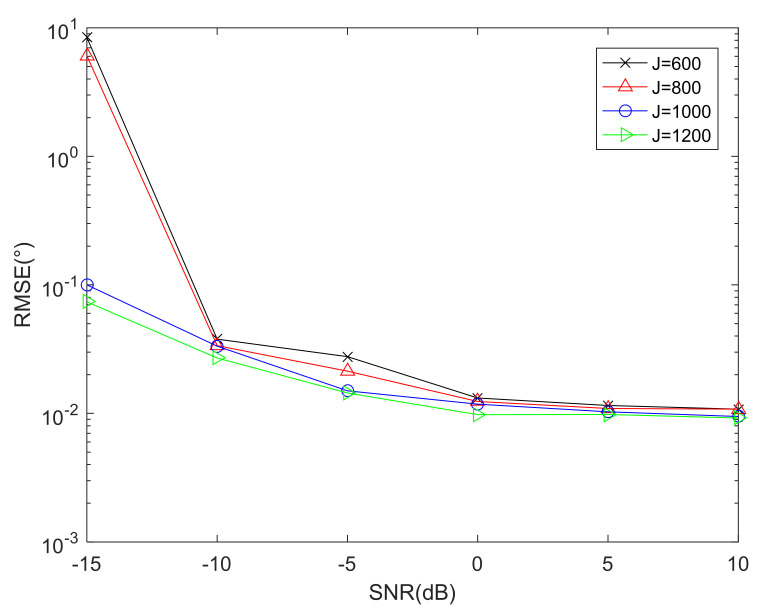
RMSE performance comparison versus snapshots.

To validate the effectiveness of the proposed array and DFT-MUSIC method in parameter estimation, Figure 6 shows the RMSE performance comparison versus snapshots with proposed array geometry by utilizing the DFT-MUSIC method, where the total number of physical sensors is T=10. As depicted in Figure 6, the increase in the number of snapshots means a more accurate FOC matrix of received signal model, which results in better DOA estimation performance. 

### 6.2. RMSE Performance Comparison versus Sensors

**Figure 7:**
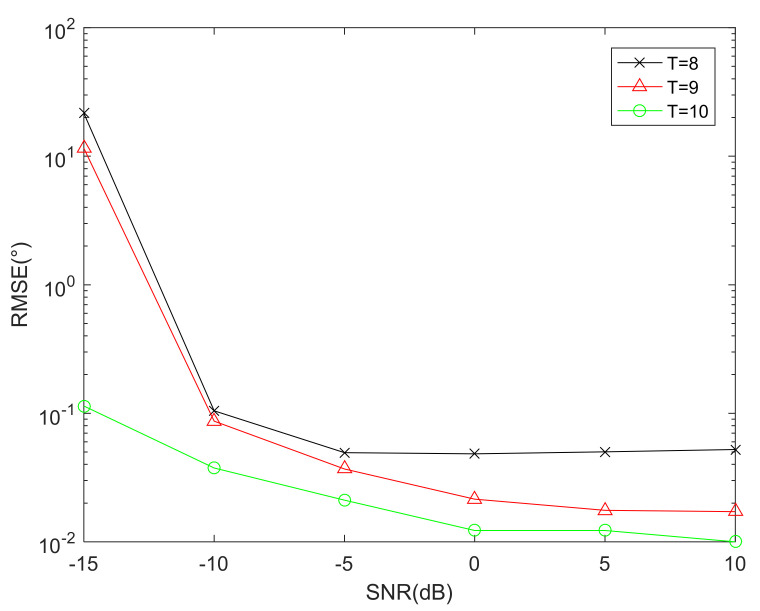
RMSE performance comparison versus sensors.

The RMSE performance comparison of the proposed array geometry versus sensors via DFT-MUSIC method is given in Figure 7. As the number of sensors increases, the diversity gains of the received signal increase. Consequently, a better DOA estimation performance can be obtained. Besides, when the total number of sensors is T=8, the location of the proposed array is {−13,−6.5,0,2.5,6.5,7.5,12.5,15}, and T=9 corresponds to {−17,−8.5,0,2.5,7.5,8.5,12.5,17.5,20}.

### 6.3. RMSE Performance Comparison of Different Arrays with DFT-MUSIC Method

**Figure 8:**
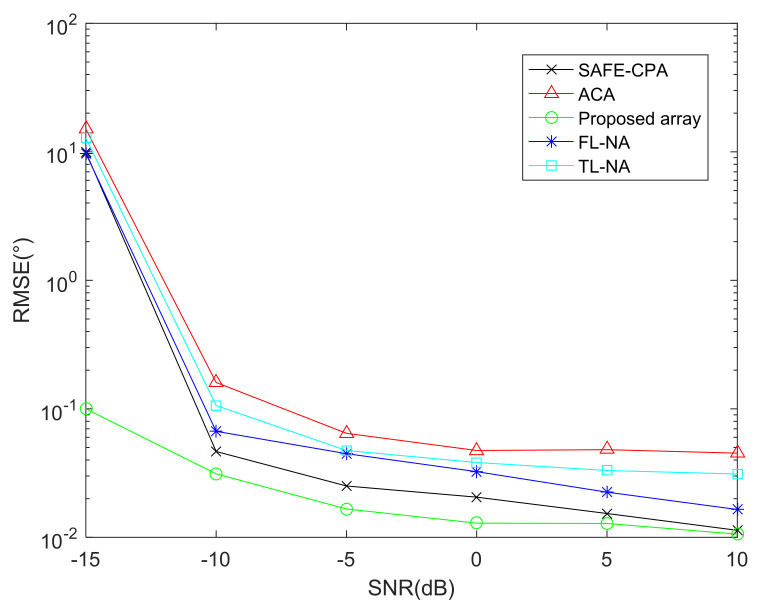
RMSE performance comparison of different arrays.

**Figure 9:**
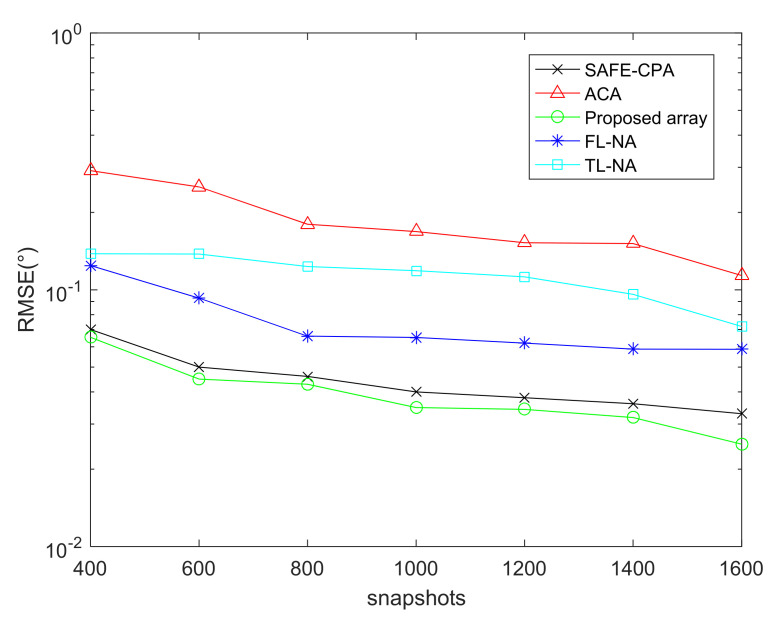
RMSE performance comparison versus snapshots.

This part depicts the RMSE performance comparison of the proposed array, ACA [14], SAFE-CPA [28], TL-NA [11], FL-NA [26] with DFT-MUSIC algorithm, where the total number of sensors is T=10. Besides, the snapshots of Figure 8 is J=1000,the SNR of Figure 9 is SNR=−10dB.

As shown in Figure 8 and Figure 9, the DOA estimation performance of all arrays has better parameter estimation performance with the increase in snapshots and SNR. In addition, considering that ACA and TL-NA are all designed based on SOC, which can be applied to vectorized FOC methods by adding 2-DC operations. Consequently, the virtual array elements locations obtained from the first 2-DC operation are not fully utilized, resulting in much redundancy. The SAFE-CPA, FL-NA and the proposed array designed based on FOC have better parameter estimation performance than the array designed based on FOC because of larger cDOF, and the proposed array achieves the maximum cDOF, which results in the best DOA estimation performance.

### 6.4. RMSE Performance Comparison of Different Algorithms

**Figure 10:**
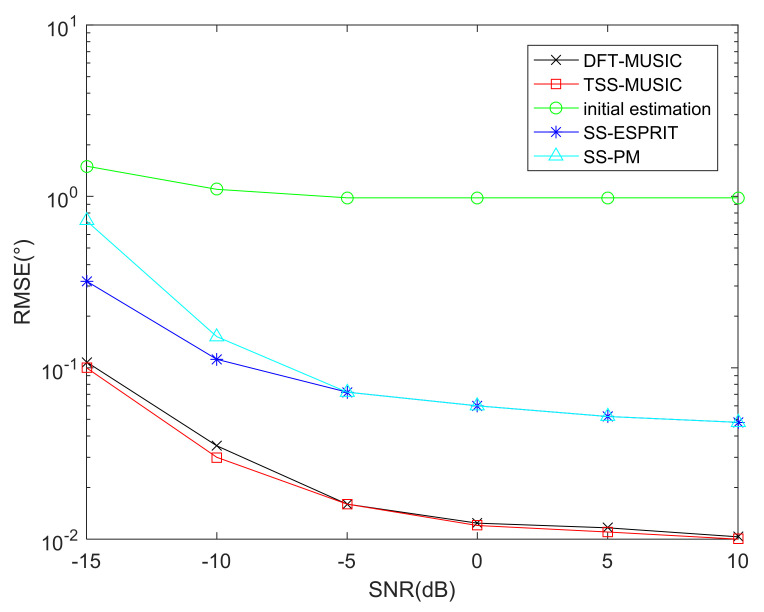
RMSE performance comparison of different algorithms.

To verify the superiority of the DFT-MUSIC method, Figure 10 shows the RMSE performance comparison of DFT-MUSIC, TSS-MUSIC, initial estimation of DFT, SS-PM and SS-ESPRIT algorithm, where the interval of searching is $\Delta =0.01^{\circ }$ and the number of snapshots is J=1000. Besides, the searching ranges of DFT-MUSIC and TSS-MUSIC are $(\theta_{k}^{ini}-1^{\circ },\theta_{k}^{ini}+1^{\circ }),k=1,\cdots K$ and $\left(-90^{\circ },90^{\circ }\right)$, respectively, and $\theta_{k}^{ini}$ represents the initial DOA estimates.

It can be concluded from Figure 10 that the DFT-MUSIC algorithm owns the same asymptotical DOA estimation performance as the TSS-MUSIC algorithm, whereas Section 5.2. points out that the computational complexity of DFT-MUSIC is much lower than the TSS-MUSIC algorithm, and the DFT-MUSIC method achieves a better balance between complexity and DOA estimation accuracy; hence, it has better application prospects.

## 7. Conclusions

In this paper, a sparse array design method for non-Gaussian signals was proposed, which was based on GPSP and the properties of UCLATS. Further, a sparse array design example was given to illustrate the procedure. The process of obtaining physical sensors location could be divided into two steps; specifically, the first step was to model the problem of obtaining the longest possible consecutive virtual 2-SC from a given number of physical array elements into a GPSP and then to utilize the solution to GPSP to obtain the position of the virtual 2-SC and the position of the initial physical array. The second step was to multiply the positions of virtual 2-SC by appropriate coefficients to generate UCLATS. Consequently, the position of physical sensors could be obtained by multiplying the corresponding coefficient to initial physical array. Besides, the DFT-MUSIC algorithm was proposed, which achieved the same parameter estimation asymptotic performance with less than 10% computational complexity as compared with the TSS-MUSIC algorithm under the same simulation conditions.

## Figures and Tables

**Table 1:** DOF of UCLATS.

M	N	${\mathrm{DOF}}_{\mathrm{UCLATS}}$
Odd	Odd	$2(N+\left\lfloor N/2\right\rfloor )M-1$
Even	Odd	$2(N+\left\lfloor N/2\right\rfloor )M-1$
Odd	Even	$2(N+\left\lfloor N/2\right\rfloor )M-(M-1)-1$
Even	Even	/

**Table 2:** The solutions to GPSP.

k	$n_{2}{(S}_{k})$		$S_{k}$											
4	8	0	1	3	4									
5	12	0	1	3	5	6								
6	16	0	1	3	5	7	8							
7	20	0	1	2	5	8	9	10						
7	20	0	1	3	4	8	9	11						
7	20	0	1	3	4	9	11	16						
7	20	0	1	3	5	6	13	14						
7	20	0	1	3	5	7	9	10						
8	26	0	1	2	5	8	11	12	13					
8	26	0	1	3	4	9	10	12	13					
8	26	0	1	3	5	7	8	17	18					
9	32	0	1	2	5	8	11	14	15	16				
9	32	0	1	3	5	7	9	10	21	22				
10	40	0	1	3	4	9	11	16	17	19	20			
11	46	0	1	2	3	7	11	15	19	21	22	24		
11	46	0	1	2	5	7	11	15	19	21	22	24		
12	54	0	1	2	3	7	11	15	19	23	25	26	28	
12	54	0	1	2	5	7	11	15	19	23	25	26	28	
12	54	0	1	3	4	9	11	16	18	23	24	26	27	
12	54	0	1	3	5	6	13	14	21	22	24	26	27	
13	64	0	1	3	4	9	11	16	21	23	28	29	31	32
⋮	⋮	⋮	⋮	⋮	⋮	⋮	⋮	⋮	⋮	⋮	⋮	⋮	⋮	⋮

**Table 3:** Comparisons of different cDOF versus array structures.

Arrays Structure	Number of Sensors *T* $\left(N_{i},i=1,2,\cdots ,4\right)$	Consecutive DOF $\left(N_{i},i=1,2,\cdots ,4\right)$	Locations (T=10)
TL-NA	$N_{1}+N_{2}$	$4N_{2}(N_{1}+1)-3$	$\left\{0.5,1,1.5,2,2.5,3,6,9,12,15\right\}$
FL-NA	$\sum_{1}^{4}N_{i}-3$	$2\prod_{i=1}^{4}N_{i}-1$	$\left\{0.5,1,1.5,2,4,6,12,18,36,54\right\}$
ACA	$2N_{1}+N_{2}-1$	$6N_{1}N_{2}+2N_{1}-2N_{2}-1$	$\left\{0,1.5,2.5,3,4.5,5,6,7.5,10,12.5\right\}$
SAFE-CPA	$2N_{1}+N_{2}-1+N_{3}$	$2M_{0}+1$	$\left\{0,1,1.5,2,3,4.5,17.5,30.5,43.5,56.5\right\}$
Proposed array	$N_{2}+N_{1}+\left\lfloor N_{1}/2\right\rfloor -2$	$\geq 2(n_{2}(S_{N_{1}})+1)(n_{2}(S_{N_{2}})+1)-1$	$\left\{-26,-19.5,-6.5,0,4.5,6.5,13,13.5,22.5,27\right\}$

$M_{0}=4N_{1}N_{2}N_{3}+3N_{1}N_{2}+2N_{1}N_{3}-N_{2}N_{3}+N_{1}-N_{2}+N_{3}-1$.

**Table 4:** cDOF examples of different array structures (T=6,⋯,10).

		*T*	6	7	8	9	10
	cDOF						
Array							
Proposed array			/	83 (5, 2, 2)	139 (5, 3, 2)	179 (6, 3, 2)	251 (5, 4, 3)
ACA			33 (2, 3)	/	53 (2, 5)	69 (3, 4)	85 (3, 5)
SAFE-CPA			/	/	137 (2, 3, 2)	189 (2, 3, 3)	241 (2, 3, 4)
TL-NA			45 (3, 3)	61 (3, 4)	77 (4, 4)	97 (4, 5)	117 (5, 5)
FL-NA			47 (3, 2, 2, 2)	71 (3, 3, 2, 2)	107 (3, 3, 3, 2)	161 (3, 3, 3, 3)	215 (4, 3, 3, 3)

**Table 5:** cDOF examples of different array structures (T=11,⋯,15).

		*T*	11	12	13	14	15
	cDOF						
Array							
Proposed array			323 (6, 4,3)	/	503 (8, 4, 3)	747 (7, 6, 4)	951 (8, 6, 4)
ACA			/	117 (4, 5)	133 (3, 8)	161 (4, 7)	177 (5, 6)
SAFE-CPA			273 (3, 4, 2)	375 (3, 4, 3)	477 (3, 4, 4)	581 (3, 5, 4)	705 (3, 5, 5)
TL-NA			141 (5, 6)	165 (6, 6)	193 (6, 7)	221 (7, 7)	253 (7, 8)
FL-NA			287 (4, 4, 3, 3)	383 (4, 4, 4, 3)	511 (4, 4, 4, 4)	639 (5, 4, 4, 4)	799 (5, 5, 4, 4)

**Table 6:** Computational complexity comparison of different algorithms.

Methods	Computational Complexity	Complex Multiplications
DFT-MUSIC	$O\{J{\left(M+N+\left\lfloor N/2\right\rfloor -1\right)}^{4}+T^{2}+W^{3}+nW(2(W-K)+1)\}$	$3.53\times 10^{7}$
TSS-MUSIC	$O\{J{\left(M+N+\left\lfloor N/2\right\rfloor -1\right)}^{4}+W^{3}+n_{1}W(2(W-K)+1)\}$	$5.87\times 10^{8}$

## Data Availability

Not applicable.

## Appendix A

(a) Considering the coprime relationship between *M* and *N*, the structure of UCLATS can be rewritten as
  (A1) $$\left\{\begin{array}{l} S_{4}=S_{1}\cup S_{3}=\langle -(N-1),\left\lfloor N/2\right\rfloor \rangle Md\\ S_{2}=\langle 0,M-1\rangle Nd \end{array}\right.$$
  (A2) $$S=S_{4}\cup S_{2}$$

Without loss of generality, suppose that M>N. According to Definition 1, the difference co-array $D_{UCLATS}$ includes the self-difference co-array set $D_{sUCLATS}$ and cross-difference co-array set $D_{cUCLATS}$, specifically,

Case 1: *N* is odd,

For the cross-difference co-array set $D_{cUCLATS}$,

(A3) $$D_{cUCLATS}=\{\pm d_{c}\left|d_{c}=Nmd-Mnd\right.\}$$

where $0\leq m\leq M-1,-(N-1)\leq n\leq \left\lfloor N/2\right\rfloor$. When m=0, the elements with $-Mnd,-\left\lfloor N/2\right\rfloor \leq n\leq \left\lfloor N/2\right\rfloor$ can form $\left\lfloor N/2\right\rfloor$ groups of values symmetric about the origin. Besides, because *M* and *N* (odd) are coprime integers, the number of distinct lags of set $\left\{d_{c}\left|d_{c}=Nmd-Mnd\right.\right\}$ is $\left(N+\left\lfloor N/2\right\rfloor )M-\left\lfloor N/2\right\rfloor \right\}$.

For the self-difference co-array set $D_{sUCLATS}$,

(A4) $$D_{sUCLATS}=\{\pm d_{s}\left|d_{s}=Nmd\right.\}\cup \{\pm d_{s}\left|d_{s}=Mnd\right.\}$$

where $0\leq m\leq M-1,0\leq n\leq (N-1)+\left\lfloor N/2\right\rfloor$. The set $\left\{\pm d_{s}\left|d_{s}=Nmd\right.\right\}$ can be considered as the specific case with n=0 in (A.3), i.e., $\{\pm d_{s}\left|d_{s}=Nmd\right.\}\subseteq D_{cUCLATS}$. Similarly, the relationship $\{\pm d_{s}\left|d_{s}=Mnd\right.\}\subseteq D_{cUCLATS}$ is established when 0≤n≤(N−1). However, for $N-1<n\leq \left\lfloor N/2\right\rfloor +(N-1)$, the set $\left\{d_{s}\left|d_{s}=Mnd\right.\right\}$ contains $\left\lfloor N/2\right\rfloor$ elements which are not included in $D_{cUCLATS}$, i.e., $\{(\left\lfloor N/2\right\rfloor +(N-1))Md,$$\left(\left\lfloor N/2\right\rfloor +(N-1)-1)Md,\cdots ,MNd\right\}$.

Based on the symmetry of 2-DC, the DOF of the UCLATS can be represented as

(A5) $$DOF_{UCLATS}=2(N+\left\lfloor N/2\right\rfloor )M-1$$

Case 2: *N* is even,

The extra distinct lags in self-difference co-array set $D_{sUCLATS}$ mentioned above is still established. It is noteworthy to analyze the cross-difference co-array set $D_{cUCLATS}$ under the condition of even *N*, where $\left\lfloor N/2\right\rfloor =N/2$. As mentioned in Case 1, the elements in $D_{cUCLATS}$ with −Mnd,−N/2≤n≤N/2 can form N/2 groups of values symmetric about the origin when m=0. Moreover, the location set $S_{3}$ can be rewritten as

(A6) $$S_{3}=\langle 0,N/2\rangle Md$$

then, the largest number in $S_{3}$ is MNd/2, However, the second to the last element of $S_{2}$ is Nd,⋯(M−1)Nd. These (M−1) lags form (M−1)/2 groups that are symmetric about MNd/2. Note that this symmetrical relationship cannot be established when *N* is odd. Thus, the number of distinct lags of set $\left\{d_{c}\left|d_{c}=Nmd-Mnd\right.\right\}$ is $\left(N+\left\lfloor N/2\right\rfloor )M-\left\lfloor N/2\right\rfloor -(M-1)/2\right\}$. Considering the discussion about $D_{sUCLATS}$, the DOF of UCLATS can be denoted as

(A7) $$DOF_{UCLATS}=2(N+\left\lfloor N/2\right\rfloor )M-(M-1)-1$$

(b) It is necessary to prove that there exists $0\leq m\leq M-1,-(N-1)\leq n\leq \left\lfloor N/2\right\rfloor$ such that $D_{UCLATS}$ contains all consecutive elements in the set [−(M+1)Nd,(M+1)Nd]. Because $D_{UCLATS}=D_{sUCLATS}\cup D_{cUCLATS}$, we consider $D_{cUCLATS}$ first, and the $D_{cUCLATS}$ contains consecutive lags in range [−(MN−1)d,(MN−1)d]. Considering the symmetry of $D_{cUCLATS}$, whose continuity of positive part can be employed to denote the whole range, which can be proved from two aspects, i.e., [0,(MN−1)d]=[0,(M−1)d]∪[Md,(MN−1)d].

For [Md,(MN−1)d], the restricted relationship 0≤m≤M−1 can be rewritten as

(A8) $$0\leq Nmd\leq N\left(M-1\right)d$$

Obviously, $Md\leq d_{c}=Nmd-Mnd\leq (MN-1)d$,i.e.,$-(MN-1)d\leq -d_{c}\leq -Md$, combined with and $Mnd=Nmd-d_{c}$, we can obtain the following relationship as

(A9) −(MN−1)d≤Mnd≤MNd−Nd−Md

which can be simplified as

(A10) $$-(N-\frac{1}{M})\leq n\leq N-\frac{N}{M}-1$$

Because of the relationship $[-(N-1),\left\lfloor N/2\right\rfloor ]\subseteq [-(N-\frac{1}{M}),N-\frac{N}{M}-1],M>N$, the integer n must satisfy the continuity with the range [Md,(MN−1)d].

For [0,(M−1)d], arbitrary elements can be obtained by different combinations of *M* and *N* with cross-difference co-array operation of S.

For [(MN+1)d,(MN+N−1)d], we take the cross-difference co-array of UCLA into consideration, which is composed of $S_{1}$ and $S_{2}$. Combing (A8) with (MN+1)d≤Nmd−Mnd≤(MN+N−1)d, the following relationship is established,

(A11) −(M+1)d≤−Mnd≤(MN+N−1)d

which can be simplified as

(A12) $$-(N+\frac{N-1}{M})\leq n\leq -\frac{M+1}{M}$$

The n must satisfy the condition −(N−1)≤n≤0. Then, the conclusion can be extended to the UCLATS (UCLA can be considered as the specific form with $S_{3}=\emptyset$). At last, the location points MNd and can be obtained by self-difference co-array of $S_{4}$, i.e., MNd=Md−(−M(N−1)d).

Generally, we have proven the positive part continuity of $D_{UCLATS}$ in range

(A13) $$\begin{array}{r} {}[0,(MN+N-1)d]=[0,(M-1)d]\cup [Md,(MN-1)d]\cup \\ MNd\cup [(MN+1)d,(MN+N-1)d] \end{array}$$

It can be also verified in Figure 4.

## Appendix B

For the convenience of presentation, Equation (21) can be rewritten as,

(A14) $${\left[{\tilde{a}}_{sort}\left(\theta_{k}\right)\right]}_{q}=\frac{1}{\sqrt{T}}\frac{\sin\left[\pi \left(q-\frac{T}{2}\sin\theta_{k}\right)\right]}{\sin\left[\frac{\pi }{T}\left(q-\frac{T}{2}\sin\theta_{k}\right)\right]}e^{-j\frac{T-1}{T}\pi \left(q-\frac{T}{2}\sin\theta_{k}\right)}$$

Considering the $\left|e^{-j\frac{T-1}{T}\pi \left(q-\frac{T}{2}\sin\theta_{k}\right)}\right|=1$, the discussions mainly focus on the value of $\frac{\sin\left[\pi \left(q-\frac{T}{2}\sin\theta_{k}\right)\right]}{\sin\left[\frac{\pi }{T}\left(q-\frac{T}{2}\sin\theta_{k}\right)\right]}$. We analyze the value of ${\left[{\tilde{a}}_{sort}\left(\theta_{k}\right)\right]}_{q}$ from two aspects, which are elaborated in the Case I and Case II below.

Besides, the value range of parameters mentioned above can be given as,

(A15) $$q\in [1,T],\frac{T}{2}\sin\theta_{k}\in [-\frac{T}{2},\frac{T}{2}],q-\frac{T}{2}\sin\theta_{k}\in \left[-\frac{T}{2}+1,\frac{3T}{2}\right],T\gg 1$$

Case I.

If $\frac{T}{2}\sin\theta_{k}=q_{k}\in Z$, considering q∈Z,then

(A16) $$\frac{\sin\left[\pi \left(q-\frac{T}{2}\sin\theta_{k}\right)\right]}{\sin\left[\frac{\pi }{T}\left(q-\frac{T}{2}\sin\theta_{k}\right)\right]}=\frac{\sin(k\pi )}{\sin(\frac{k}{T}\pi )}$$

where $k=q-\frac{T}{2}\sin\theta_{k}\in \left[-\frac{T}{2}+1,\frac{3T}{2}\right]$, and obviously,

(A17) $$\sin(k\pi )=\sin(\frac{k}{T}\pi )=0,\frac{k}{T}\in \left[-\frac{1}{2}+\frac{1}{T},\frac{3}{2}\right]$$

Actually, the value of $\frac{k}{T}$ can be determined by (A17),

(A18) $$\frac{k}{T}=0\;\;or\;\;\frac{k}{T}=1$$

For $\frac{k}{T}=0\;$,

(A19) $$\frac{\sin(k\pi )}{\sin(\frac{k}{T}\pi )}=T,e^{-j\frac{T-1}{T}\pi (q-\frac{T}{2}\sin\theta_{k})}=1$$

For $\frac{k}{T}=1$,

(A20) $$\frac{\sin(k\pi )}{\sin(\frac{k}{T}\pi )}=T\cdot {\left(-1\right)}^{T-1},e^{-j\frac{T-1}{T}\pi (q-\frac{T}{2}\sin\theta_{k})}={\left(-1\right)}^{T-1}$$

Thus, the value of $q_{k}$-th of ${\tilde{a}}_{sort}\left(\theta_{k}\right)$ is

(A21) $${\left[{\tilde{a}}_{sort}\left(\theta_{k}\right)\right]}_{q=q_{k}}=\sqrt{T}$$

where $q=q_{k}=\frac{T}{2}\sin\theta_{k}$.

When $q\neq q_{k},q_{k},q\in Z$,

(A22) $$\sin(\frac{k}{T}\pi )\neq 0,\frac{\sin(k\pi )}{\sin(\frac{k}{T}\pi )}=0\Rightarrow {\left[{\tilde{a}}_{sort}\left(\theta_{k}\right)\right]}_{q}=0$$

Case II.

If $\frac{T}{2}\sin\theta_{k}=q_{k}\notin Z$, considering q∈Z, then

(A23) $$\frac{\sin\left[\pi \left(q-\frac{T}{2}\sin\theta_{k}\right)\right]}{\sin\left[\frac{\pi }{T}\left(q-\frac{T}{2}\sin\theta_{k}\right)\right]}=\frac{\sin(k\pi )}{\sin(\frac{k}{T}\pi )}$$

Obviously, $k,\frac{k}{T}\notin Z$,i.e., $\frac{\sin(k\pi )}{\sin(\frac{k}{T}\pi )}\neq 0$. Based on the discussion of Case I, if $q=round\left\{q_{k}\right\}$, the value of $\left|q-q_{k}\right|\approx 0$(T≫1). Then, the value of (A23) is close to $\sqrt{T}$.

When $q\neq round\left\{q_{k}\right\},q\in Z,q_{k}\notin Z$, considering the relationship

(A24) $$\left|\frac{\sin(k\pi )}{\sin(\frac{k}{T}\pi )}\right|\leq 1,\left|e^{-j\frac{T-1}{2}\left(\frac{2\pi }{T}q-\pi \sin\theta_{k}\right)}\right|=1,T\gg 1$$

The ${\left[{\tilde{a}}_{sort}\left(\theta_{k}\right)\right]}_{q}$ will be a small value.
